# Intra-articular magnesium to alleviate postoperative pain after arthroscopic knee surgery: a meta-analysis of randomized controlled trials

**DOI:** 10.1186/s13018-021-02264-1

**Published:** 2021-02-05

**Authors:** Lijun Shi, Haiyun Zhu, Jinhui Ma, Li-Li Shi, Fuqiang Gao, Wei Sun

**Affiliations:** 1 Department of Orthopedic, Surgery Graduate School of Peking Union Medical College, China-Japan Friendship institute of Clinical Medicine, 2 Yinghuadong Road, Chaoyang District, 100029 Beijing, China; 2 Department of Intensive Care Unit, Tianjin Academy of Traditional Chinese Medicine Affiliated Hospital, 354 North Road, Hongqiao District, Tianjin, 300120 China; 3 Department of Orthopaedic Surgery, China-Japan Friendship Hospital, 2 Yinghuadong Road, Chaoyang District, Beijing, 100029 China; 4 Department of Gastroenterology, Henan Provincial People’s Hospital, Weiwu road No 7, Jinshui district, Zhengzhou City, 450003 Henan province China

**Keywords:** Intra-articular, Magnesium, Arthroscopic knee surgery, Postoperative pain, meta-analysis

## Abstract

**Objective:**

We aimed to evaluate the safety and efficacy of intra-articular (IA) magnesium (Mg) for postoperative pain relief after arthroscopic knee surgery.

**Methods:**

We searched PubMed, Embase, Medline, Cochrane library, and Web of Science to identify randomized controlled trials that compared postoperative pain outcomes with or without IA Mg after knee arthroscopy. The primary outcomes were pain intensity at rest and with movement at different postoperative time points and cumulative opioid consumption within 24 h after surgery. Secondary outcomes included the time to first analgesic request and side effects.

**Results:**

In total, 11 studies involving 677 participants met the eligibility criteria. Pain scores at rest and with movement 2, 4, 12, and 24 h after surgery were significantly lower, doses of supplementary opioid consumption were smaller, and the time to first analgesic requirement was longer in the IA Mg group compared with the control group. No significant difference was detected regarding adverse reactions between the groups.

**Conclusions:**

Intra-articular magnesium is an effective and safe coadjuvant treatment for relieving postoperative pain intensity after arthroscopic knee surgery.

Protocol registration at PROSPERO: CRD42020156403.

## Introduction

Arthroscopic knee surgery is an established orthopedic procedure that is performed for diagnostic and therapeutic purposes for intra-articular lesions. It has replaced classic arthrotomy in many cases due to its smaller surgical incision, fewer complications, and faster recovery [1, 2]. However, this procedure is sometimes associated with moderate to acute postoperative pain, which may hinder early mobilization and rehabilitation, and prolong hospital stays; all of which affect patient satisfaction. Therefore, it is essential to strengthen postoperative pain management and enhance convalescence after surgery.

Currently, various strategies have been introduced for the early postoperative pain management after arthroscopic knee surgery, including oral opioid analgesics, intravenous patient-controlled analgesia, and peripheral nerve blocks [3]. Neuraxial blocks such as spinal or epidural analgesia are no longer the first choice for fast-track arthroscopic surgery because of their various side effects, including headache, epidural hematoma, urinary retention, and prolonged motor block. Recent studies have recommended intra­articular (IA) drug administration for pain control due to their ability to directly block nociceptive stimuli at the local site, with less systemic absorption [4, 5]. Commonly used IA drugs in clinical practice include opioids (morphine, pethidine, fentanyl, and sufentanil), corticosteroids, clonidine, ketorolac, and local anesthetics (bupivacaine, levobupivacaine, lignocaine, lignocaine, and ketamine) [6–10]. A relatively new approach is the use of IA magnesium (Mg), which recently has been studied extensively.

Mg plays an important role in maintaining organismal homeostasis, and it is also a crucial element for cellular signal transduction [11]. Animal studies have demonstrated that Mg can alter the duration and perception of pain as it antagonizes *N*-methyl-d-aspartate (NMDA) receptors [12]. NMDA receptors not only participate in central sensitization, modulation, and nociceptive transmission of acute pain [13] but also correlate with the peripheral sensory transmission of noxious signals. In addition to their central location, NMDA receptors are also located within the peripheral skin [14], muscles [15], and the knee joint [16], where they contribute to human pain after activation [15]. At resting states without stimulus, NMDA receptors are blocked by the presence of Mg ions. Upon receiving afferent activities, nociceptor fibers dislodge Mg ions from the NMDA receptor, activating nociceptors to produce pain.

Clinically, the identified routes of Mg administration for postoperative pain control include intrathecal, epidural, systemic, and topical use, which result in different effects [17–19]. Among these routes, the IA route is likely to be more acceptable for patients due to its intrinsic safety and minimal side effects. Although a large number of clinical studies have been performed to determine the effects of IA Mg administration on postoperative pain outcomes, the findings remain controversial [20–22].

Therefore, the major objective of this quantitative meta-analysis of randomized controlled trials (RCTs) was to investigate the effect of IA Mg on acute pain management outcomes after arthroscopic knee surgery. A secondary aim was to evaluate possible side effects related to the administration of IA Mg.

## Methods

We performed this meta-analysis in accordance with the guidelines of the Preferred Reporting Items for Systematic Reviews and Meta-Analysis (PRISMA) [23].

### Literature search

Three authors (Lijun Shi, Haiyun Zhu, and Jinhui Ma) independently searched (first by title and abstract, and then by full text) the electronic databases PubMed, Medline, Embase, Web of Science, and Cochrane library from inception until October 30, 2020. The words and MESH terms “Intra-Articular,” “Magnesium,” “Arthroscopy,” “Postoperative,” and “Pain” were searched individually and in different combinations. A manual search of references from eligible and relevant studies was performed to find additional trials. No restrictions were imposed regarding language or publication status.

### Inclusion and exclusion criteria

Eligible studies were required to meet the following inclusion criteria: (1) RCTs, (2) participants undergoing arthroscopic knee surgery, (3) administration of Mg through the IA route, (4) including an experimental group of IA Mg or IA Mg plus a local anesthetic, and (5) including a control group of saline or local anesthetic alone. The exclusion criteria were as follows: (1) non-RCTs; (2) reviews, letters, abstracts, case series, or editorials; (3) the administration of Mg not through the IA route; and (4) studies with insufficient data.

### Study selection

Two authors (Lijun Shi and Lili Shi) independently assessed the initial search results to exclude irrelevant trials and identify eligible studies according to the inclusion and exclusion criteria by screening titles and abstracts. Full texts of any potentially useful studies were reviewed. Any discrepancies were resolved by consulting with a third author (Wei Sun or Fuqiang Gao).

### Data abstraction

Two authors (Lijun Shi and Haiyun Zhu) independently evaluated the included studies and extracted trial details using special data collection forms developed for this investigation. Disagreements were resolved by consensus or consultation with a third author (Wei Sun or Fuqiang Gao).

We first extracted data from tables or text. For data not reported numerically, we extracted them from available figures using the software GetData (http://getdata-graph-digitizer.com/index.php). Continuous data were reported using means and standard deviations (SD), and data presented in terms of the median and range were converted to means and SD [24]. For trials that involved more than one experimental group in comparison with a single control group, the relevant comparisons to the comparator were split for primary analysis.

The data extracted from trials included the first author, year of publication, sample size, patient baseline characteristics, type of surgery, type of anesthesia, IA Mg dose, pain scores at rest and with movement (postoperative 2, 4, 12 and 24 h), cumulative opioid consumption, time to first rescue analgesic request (min), and adverse events. The pain intensity was measured using the 10-point visual analog scale (VAS), where 0 means no pain and 10 means the most severe pain. The numerical rating scale (NRS) of pain was converted to a VAS score. Postoperative opioid consumption within 24 h was converted to the equivalent dosage of intravenous (IV) morphine [25].

The primary outcomes of interest were the pain VAS scores at rest and with movement at different postoperative time points and total opioid consumption (IV morphine equivalent, mg) in the first 24-h postoperative period. The secondary outcomes included the time to first analgesic requirement (min) and the incidence of side effects.

### Assessments of the risk of bias and methodological quality

Two senior authors (Fuqiang Gao and Wei Sun) independently evaluated the methodological quality of the included studies using the Cochrane Collaboration’s Risk of bias tool [26], which contains seven domains: random sequence generation, allocation concealment, blinding of participants and personnel, blinding of outcome assessment, incomplete outcome data, selective outcome reporting, and other sources of bias. The risk of bias was defined as high, low, and unclear. Disagreements were resolved by discussion.

The quality of evidence for each outcome was judged with the Grading of Recommendations Assessment, Development and Evaluation (GRADE) methodology [27], which consists of five items: study limitations, inconsistency of results, indirectness of evidence, imprecision, and reporting bias. This methodology categorizes the strength of evidence as high, moderate, low, or very low, and each of these items may be used to define the quality level. This process was conducted using Grade Profiler software (GRADEpro version 3.6).

### Statistical analysis

All statistical analyses were conducted using Review Manager software (RevMan version 5.3). Continuous variables are reported as mean differences (MD) with 95% confidence intervals (CIs). As the incidence of adverse events was very low, only qualitative analysis and description was performed. Statistical heterogeneity was measured and reported as *I*^2^, which describes the percentage of the total variability caused by heterogeneity rather than by chance. The *I*^2^ values ranged between 0 and 100%, where values above 50 and 75% represent substantial and considerable heterogeneity, respectively. If the heterogeneity was significant (*p* < 0.05, *I*^2^ > 50%), the random-effects model was used. Otherwise, the fixed-effects model was adopted (*p* > 0.05, *I*^2^ < 50%). Sensitivity analysis was further performed by removing one trial at a time to explore possible explanations for heterogeneity and to identify the influence of a single RCT on the overall mean differences.

## Results

### Search results and selected articles

**Fig. 1:**
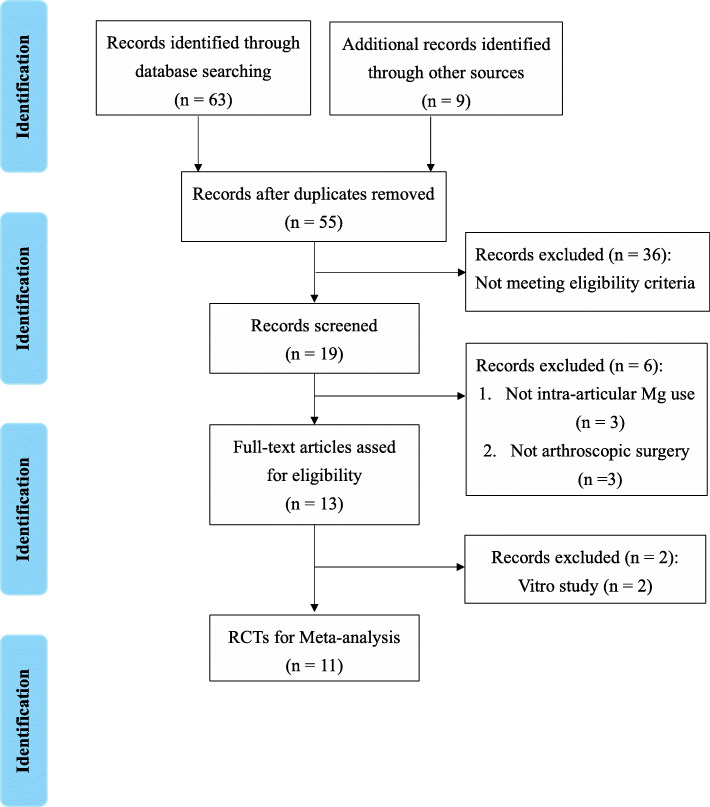
Flow chart of the randomized controlled trials selection process

Figure 1 illustrates the screening process of literature search. Finally, from the retrieved studies, 11 (published between 2006 and 2018) [28–38] met the inclusion criteria and were qualified for this meta-analysis. The main characteristics of the included studies (including 677 participants) are summarized in Table 1. Five studies compared IA Mg versus saline or bupivacaine alone [28, 30, 32, 33, 36], five other studies compared IA Mg plus bupivacaine versus bupivacaine [29, 31, 34, 35, 38], while one study contained these two kinds of comparisons [37], and both were included. All studies were of RCT design with individual sample sizes ranging from 18 to 51.

**Table 1:** Main characteristics of the 11 included studies

Author, year	Country	Groups (n)	Interventions	Age (y), %Males	Type of arthroscopy	Anesthesia	Main outcomes
Sadoni, 2017 [28]	Iran	Mg: 51	Mg 1 g + saline to 20 ml	30.6 ± 7.6, 82.3%	Meniscectomy	General	VAS, AC, AD
		P: 43	Saline 20 ml	29.9 ± 9.5, 79.0%			
Devi, 2018 [29]	India	Mg + B: 18	Mg 10 mg/kg + 0.25% B to 20 ml	33.44 ± 10.87, 72.2%	Arthroscopy	Spinal	VAS, AC
		B: 18	0.25% B 20 ml	37.22 ± 13.36, 55.5%			
Abdulatif, 2015 [30]	Egypt	Mg: 28	Mg 1 g + saline to 20 ml	34.6 ± 9.3, 60.7%	ACL reconstruction	FNB + General	VAS, AC, AD, SE
		P: 27	Saline 20 ml	35.0 ± 10.4, 74.0%			
FAROUK, 2009 [31]	Egypt	Mg + B: 20	Mg 0.15 g + 0.25% B to 20 ml	36 ± 6, 90%	Meniscectomy	General	VAS, AC, AD
		B: 20	0.25% B 20 ml	35 ± 7, 80%			
Radwan, 2012 [32]	Egypt	Mg: 20	Mg 0.8 g + saline to 20 ml	31.10 ± 7.90, 85%	Meniscectomy	General	VAS, AC, AD, SE
		B: 20	0.5% B 20 ml	32.20 ± 9.62, 90%			
Koltka, 2011 [33]	Turkey	Mg: 30	Mg 0.5 g + saline to 20 ml	48.4 ± 11, 30%	Meniscectomy	General	NRS, AC, AD
		P: 30	Saline 20 ml	46.0 ± 15.6, 36.6%			
Suhrita, 2009 [34]	Kolkata	Mg + B: 30	Mg 0.5 g+ 0.25% B to 20 ml	37.9 ± 11.8, 36.6%	Meniscectomy, Ligament repair	General	VAS, AC, AD, SE
		B: 30	0.25% B + saline to 20 ml	36.8 ± 10.6, 70%			
Kizilcik, 2017 [35]	Turkey	Mg + LB: 32	Mg 1.5 g + LB 50 mg to 15 ml	43.06 ± 13.19, 68.7%	Meniscectomy	General	VAS, AC, SE
		LB: 32	LB 100 mg 10 ml	40.06 ± 9.24, 75%			
Bondok, 2006 [36]	Egypt	Mg: 30	Mg 0.5 g + saline to 10 ml	27 ± 4, 100%	Meniscectomy	General	VAS, AC, AD
		P: 30	Saline 10 ml	25 ± 4, 100%			
Elsharnouby, 2008 [37]	Egypt	Mg: 27 P: 27	Mg 1 g + saline to 20 ml Saline 20 ml	39 ± 12, 11.1% 41 ± 13, 14.8%	Meniscectomy	General	VAS, AC, AD
		Mg + B: 27 B: 27	Mg 1 g + 0.25% B to 20 ml 0.25% B 20 ml	40 ± 11, 3.7% 45 ± 9, 7.4%			
Venkateshamurthy, 2018 [38]	India	Mg + B: 30	Mg 1 g + 0.25% B to 30 ml	Un	Arthroscopy	General	VAS, AC, AD
		B: 30	0.5% B + saline to 30 ml				

*Mg* magnesium sulfate, *B* bupivacaine, *P* placebo, *LB* levobupivacaine, *ACL* anterior cruciate ligament, *Un* unknown, *FNB* femoral nerve block, *VAS* visual analog scale score, *NRS* numeric rating scale, *AC* analgesic consumption, *AD* analgesic consumption duration, *SE* side effects

### Study quality and GRADE of evidence

**Fig. 2:**
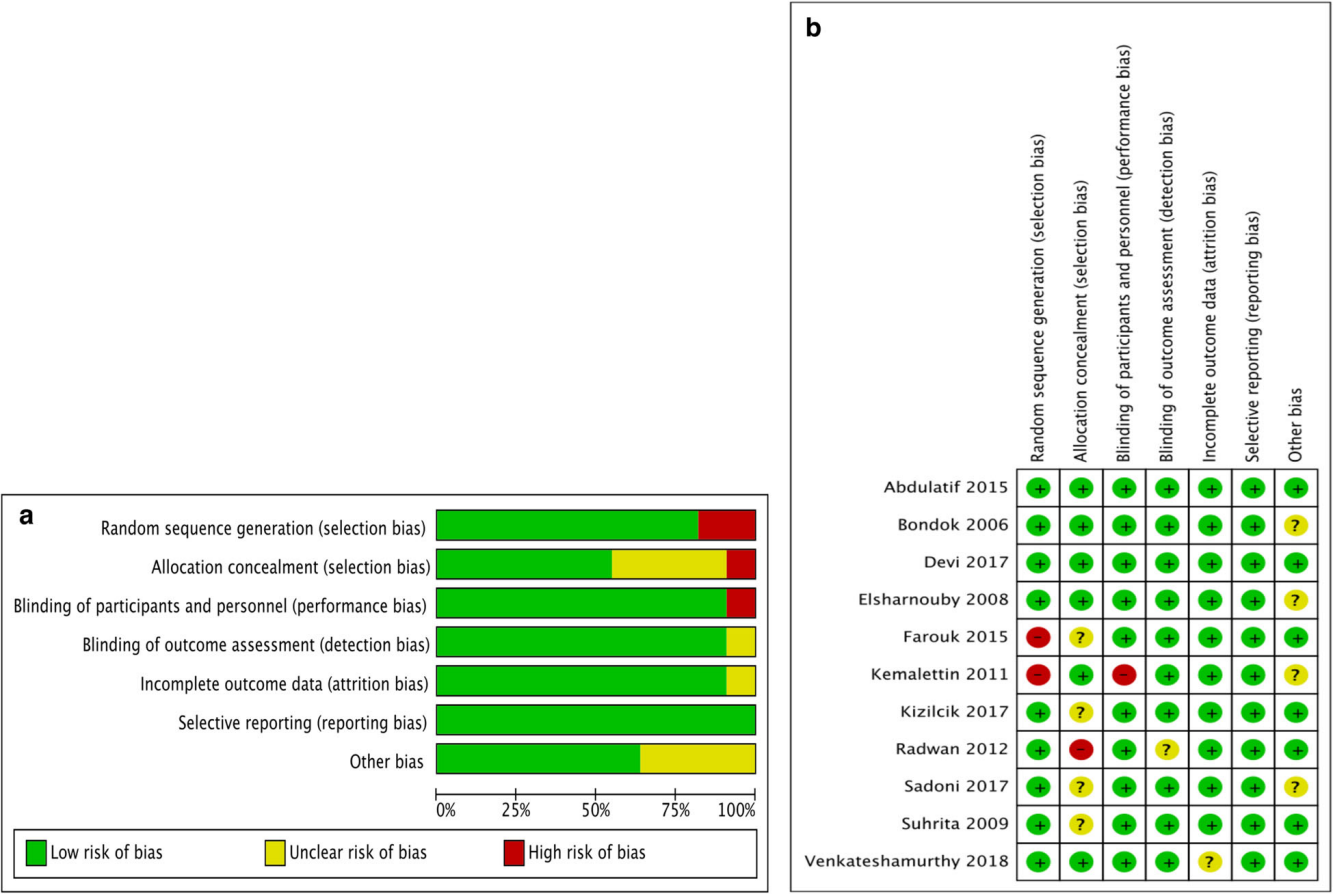
Risk of bias assessment for the included randomized controlled trials

Figure 2 is a summary of the risk of bias assessment. Two studies [30, 31] did not describe their random sequence generation (high risk of selection bias) and four [28, 31, 34, 35] did not design a clear allocation concealment plan (unclear or high risk of selection bias). All trials adopted the double-blind method, except one [33], which adopted a single-blind method (high risk of performance bias). The GRADE level of evidence for each RCT is shown in Table 2, and the quality was mostly high or moderate.

**Table 2:** The GRADE evidence quality for each outcome

Outcome	Number of Included Studies	Total Participants (Mg/ Control)	MD (95% CI)	Heterogeneity	Quality of Evidence (GRADE)
VAS at rest					
at 2h	8	212/211	-0.74 (-0.84, -0.64)	I^2^ = 0%, P = 0.51	LOW
at 4h	6	152/151	-0.24 (-0.37, -0.11)	I^2^ = 45%, P = 0.11	MODERATE ^d^
at 12h	6	152/152	-0.53 (-0.64, -0.41)	I^2^ = 47%, P = 0.10	HIGH
at 24h	7	186/186	-0.33 (-0.42, -0.24)	I^2^ = 30%, P = 0.20	HIGH
VAS with movement					
at 2h	7	140/139	-0.46 (-0.64, -0.27)	I^2^ = 39%, P = 0.14	HIGH
at 4h	6	150/149	-0.85 (-1.40, -0.30)	I^2^ = 95%, P <0.00001	MODERATE ^b^
at 12h	6	150/149	-0.83 (-1.17, -0.48)	I^2^ = 71%, P = 0.004	MODERATE ^c^
at 24h	7	170/169	-0.58 (-0.79, -0.36)	I^2^ = 45%, P = 0.09	HIGH
Anesthetic Consumption	8	229/220	-4.23 (-4.64, -3.82)	I^2^ = 27%, P = 0.21	HIGH
Anesthetic duration	11	311/302	329.99 (228.73, 431.24)	I^2^ = 99%, P<0.00001	LOW ^b^

(1) GRADE working group grades of evidence:

*High quality*: Further research is very unlikely to change our confidence in the estimate of effect

*Moderate quality*: Further research is likely to have an important impact on our confidence in the estimate of effect and may change the estimate

*Low quality*: Further research is very likely to have an important impact on our confidence in the estimate of effect and is likely to change the estimate

*Very low quality*: Any estimate of effect is very uncertain

(2) Explanations:

^a^Study limitation: included trials are quasi design

^b^Inconsistency of results: large heterogeneity

^c^Indirectness of evidence: large differences between the interventions in different trials

^d^Imprecision: small sample size and wide 95%CI

^e^Reporting bias: positive values showing benefits of the studied intervention

### Meta-analysis results

#### VAS scores at rest

**Fig. 3:**
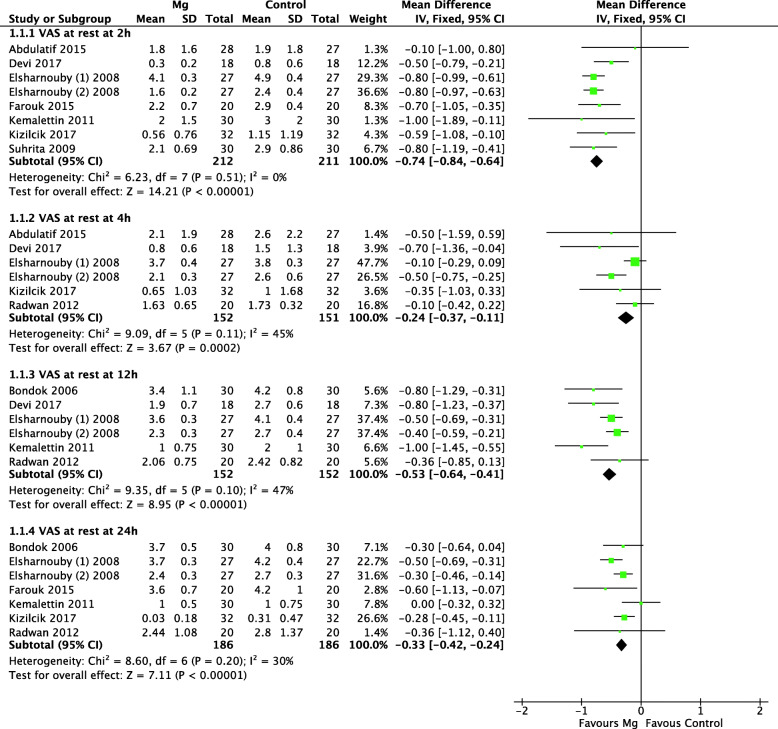
Forest plots of the meta-analysis that compared VAS scores at rest at postoperative 2, 4, 12, and 24 h

The pooled effects of IA Mg on postoperative pain after arthroscopic knee surgery are summarized in Table 2. Nine studies, including ten trials, compared the pain intensity at rest between IA Mg participants and non-IA Mg participants. As shown in Fig. 3, IA Mg was associated with significantly lower VAS scores at postoperative 2 h (MD = − 0.74, 95% CI: − 0.84 to − 0.64; *p* = 0.51; *I*^2^ = 0%), 4 h (MD = − 0.24, 95% CI: − 0.37 to − 0.11; *p* = 0.11; *I*^2^ = 45%), 12 h (MD = − 0.53, 95% CI: − 0.64 to − 0.41; *p* = 0.10; *I*^2^ = 47%), and 24 h (MD = − 0.33, 95% CI: − 0.42 to − 0.24; *p* = 0.20; *I*^2^ = 30%) (Table 2). The heterogeneity was all acceptable; hence, a fixed-effects model was used, and further sensitivity analysis was not performed.

#### VAS scores with movement

**Fig. 4:**
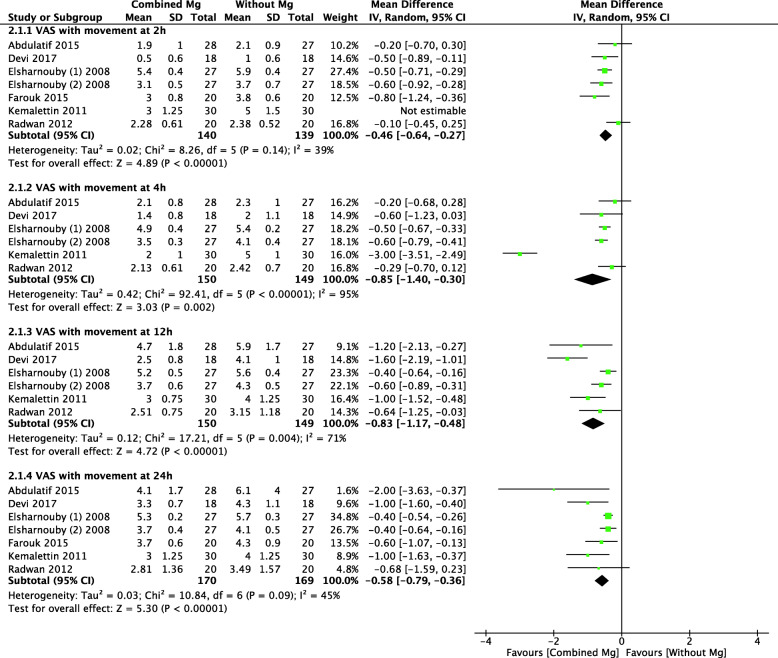
Forest plots of the meta-analysis that compared VAS scores with movement at postoperative 2, 4, 12, and 24 h

Seven trials compared the postoperative pain intensity with movement between two groups. As shown in Fig. 4, IA Mg was associated with significantly lower VAS scores at postoperative 2 h (MD = − 0.46, 95% CI: − 0.64 to − 0.27; *p* = 0.14; *I*^2^ = 39%), 4 h (MD = − 0.85, 95% CI: − 1.40 to − 0.30; *p* < 0.0001; *I*^2^ = 95%), 12 h (MD = − 0.83, 95% CI: − 1.17 to − 0.48; *p* = 0.004; *I*^2^ = 71%), and 24 h (MD = − 0.58, 95% CI: − 0.79 to − 0.36; *p* = 0.05; *I*^2^ = 45%) (Table 2). But the heterogeneity was significant at postoperative 4 h, sensitivity analysis demonstrated that removal of the study by Kemalettin et al. [33] significantly changed the results (MD = − 0.51, 95% CI: − 0.62 to − 0.39; *p* = 0.45; *I*^2^ = 0%). In this RCT, postoperative analgesia was maintained by IV tramadol during the first 4 h after surgery. Meanwhile, sensitivity analyses after excluding one trial at a time still showed a substantial heterogeneity in the pain outcomes at postoperative 12 h.

#### Postoperative opioid consumption

**Fig. 5:**
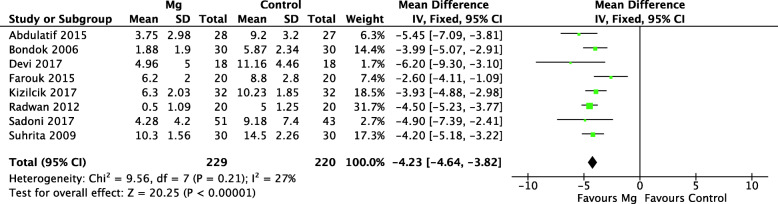
Forest plots of the meta-analysis that compared morphine consumption within the 24-h postoperative timeframe

Eight trials compared the postoperative opioid consumption (IV morphine equivalent) between two groups. As shown in Fig. 5, IA Mg was associated with significantly less opioid consumption within postoperative 24 h (MD = − 4.23, 95% CI: − 4.64 to − 3.82; *p* = 0.21; *I*^2^ = 27%) (Table 2). No statistical heterogeneity was observed, and a fixed-effects model was used.

#### Time to first analgesic request (min)

**Fig. 6:**
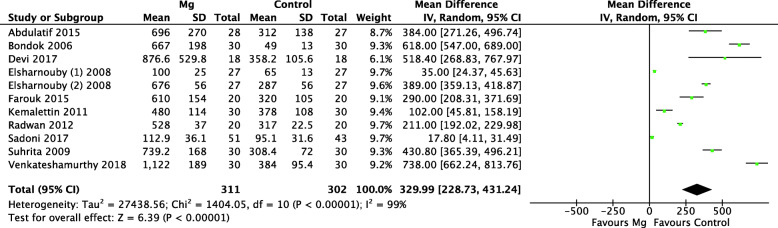
Forest plots of the meta-analysis that compared the time to first analgesic request within the 24-h postoperative timeframe

Eleven trials compared the time to first analgesic request after surgery between two groups. As shown in Fig. 6, IA Mg was associated with significantly prolonging of the time to analgesic requirement (MD = 329.99, 95% CI: 228.73–431.24; *p* < 0.00001; *I*^2^ = 99%) (Table 2). The heterogeneity was considerable; however, further sensitivity analysis did not change the heterogeneity when any of the studies were removed.

#### Safety analysis

Only three included RCTs reported adverse reactions. In the study by Abdulatif et al. [30], postoperative shivering was observed in 12 and 10 patients in the IA Mg administration (*n* = 28) and control (*n* = 27) groups, respectively. In the RCT conducted by Radwan et al. [32], one patient in both the IA Mg (*n* = 20) and placebo (*n* = 20) groups developed knee effusion. In the study conducted by Suhrita et al. [34], two patients developed hypotension and bradycardia in the IA Mg group (*n* = 30), while no side effects were observed in the placebo group (*n* = 30). There was no statistically significant difference between the comparable groups in each RCT.

## Discussion

The chief finding of this study was that IA Mg can significantly relieve pain intensity within the 24-h postoperative timeframe after arthroscopic knee surgery. The VAS scores at rest or with movement were lower in the IA Mg group than in the non-IA Mg group. Furthermore, the IA Mg group showed lower postoperative opioid consumption and the time to first analgesic request after surgery was longer, which would help reduce the risk of opioid-related complications. Though three articles reported adverse reactions, there was no statistically significant difference between the comparable groups. Currently, IA Mg is not considered a standard strategy for postoperative pain control. However, these results of this meta-analysis based on 11 clinical RCTs provide strong evidence that IA Mg is useful and safe. This method can be an effective coadjuvant treatment for postoperative pain management after arthroscopic knee surgery.

Mg has been used widely for many indications. As to the analgesic effect, Mg does not possess direct analgesic activity, as its function primarily relies on its role as physiological NMDA receptor antagonists [11]. Nociceptive sensitization of pain stimuli requires calcium for the release of neurotransmitters and other substances. The potential mechanism of the antinociceptive effects of Mg may be that Mg blocks the calcium channel in a voltage-dependent way. Mg can produce a dramatic reduction of NMDA-induced currents. In the knee joint, NMDA receptors are located not only in the peripheral termini of primary afferent fibers but also in cellular elements such as immune cells and synoviocytes [39]. Therefore, it is possible that local Mg administration could provide analgesic effects through an IA route.

Furthermore, Mg also has other beneficial biological effects. Some research showed that adding Mg to a local anesthetic can reduce toxic effect of the latter to articular chondrocytes [40]. Besides, local Mg administration could recruit endogenous stem cells and promote fibrocartilaginous matrix synthesis, promoting in situ meniscal repair [41]. Mg deficiency in the extracellular matrix of a cartilage may lead to typical joint cartilage lesions [42]. In contrast, high concentrations of IA Mg can significantly inhibit extracellular matrix calcification and protect articular cartilage [43, 44]. Similarly, clinical trials have found that subjects with lower levels of serum Mg had a higher prevalence of knee chondrocalcinosis [45]. Further study is still needed to clarify the mechanism of the effect of magnesium.

The results of this study are in accordance with the findings of several published RCTs with reasonable design and adequate follow-up time [46–48]. Although Mg were used in different ways in clinical practice, the above evidences indicate an overall beneficial effect of Mg on postoperative pain relief. This strategy is suitable not only for arthroscopic knee surgery but also for multiple orthopedic surgeries. Better pain control at early postoperative stage may accelerate the rapid recovery and functional rehabilitation after joint surgery.

Concerns about Mg-related complications still remain. Three trials reported adverse reactions in this meta-analysis. Postoperative shivering is a common manifestation after anesthesia, which can lead to perioperative ischemia [49]. However, Gildasio et al. reported that perioperative systemic Mg can reduce the incidence rates of postoperative shivering [50]. Another concern is the increased risk of infection, as previous studies have shown that preoperative IA injections increase the risk of infection after total knee arthroplasty [51], especially corticosteroid or hyaluronic acid injection within 3 months of total knee arthroplasty [52]. However, different from IA injection prior to total knee arthroplasty, IA Mg injection during knee arthroscopy is much safer due to its simplicity, short operative time, and rigid aseptic technique. More importantly, IA injections of Mg can attenuate osteoarthritis progression and suppress synovial inflammation [53]. Moreover, no relevant joint infections have been reported in clinical trials.

### Strengths and limitations

This is the first study that examined the analgesic effects of IA Mg after arthroscopic surgery, and the results demonstrated its efficacy and safety. The findings have important clinical implication, providing a novel strategy for the pain management. Then, the literature search is thorough and comprehensive, and the included studies are all eligible RCTs, which are considered the greatest level of evidence. Overall, the methodological quality of included studies is moderate or high. All these strengths may ensure the accuracy and reliability of findings.

The limitations of this study should also be acknowledged. First, this meta-analysis included 11 eligible studies (12 trials), 5 trials compared Mg with saline alone and the rest compared Mg plus analgesic with analgesic alone. The former was not sufficiently rigorous because single saline injection was not a standard clinical practice for analgesia, and it was used as only a placebo in these cases. Second, the heterogeneity was high for some outcomes, which could affect the results. After careful analysis, we found that the different types of surgery, anesthesia, IA drugs, and data recording methods may all account for the heterogeneity. Finally, the dosages of IA Mg were different with a large range in each group. It is difficult to determine the optimal dosage to truly evaluate the safety of IA Mg administration.

## Conclusions

In conclusion, the current results suggest that IA Mg can significantly reduce the pain intensity and reduce additional analgesic consumption after arthroscopic knee surgery within postoperative 24 h. This strategy appears as an effective and safe coadjuvant treatment for postoperative pain control after arthroscopic knee surgery.

## Data Availability

The datasets generated/analyzed during the current study are available.

## Abbreviations

IA: Intra-articular Mg Magnesium NMDA *N*-Methyl-d-aspartate VAS Visual analog scale NRS Numerical rating scale IV Intravenous RCT Randomized controlled trial PRISMA Preferred Reporting Items for Systematic Reviews and Meta-Analyses GRADE Grading of Recommendations Assessment, Development and Evaluation SD Standard deviation MD Mean differences CI Confidence interval *I*^2^ Statistical heterogeneity *N* Sample size
